# Perceived publication pressure in Amsterdam: Survey of all disciplinary fields and academic ranks

**DOI:** 10.1371/journal.pone.0217931

**Published:** 2019-06-19

**Authors:** Tamarinde L. Haven, Lex M. Bouter, Yvo M. Smulders, Joeri K. Tijdink

**Affiliations:** 1 Department of Philosophy, Vrije Universiteit, Amsterdam, North Holland, The Netherlands; 2 Department of Epidemiology and Biostatistics, Amsterdam UMC, location VUmc, Amsterdam, North Holland, The Netherlands; 3 Department of Internal Medicine, Amsterdam UMC, location VUmc, Amsterdam, North Holland, The Netherlands; 4 Department of Medical Humanities, Amsterdam UMC, location VUmc, Amsterdam, North Holland, The Netherlands; Mayo Clinic, UNITED STATES

## Abstract

Publications determine to a large extent the possibility to stay in academia (“publish or perish”). While some pressure to publish may incentivise high quality research, too much publication pressure is likely to have detrimental effects on both the scientific enterprise and on individual researchers. Our research question was: What is the level of perceived publication pressure in the four academic institutions in Amsterdam and does the pressure to publish differ between academic ranks and disciplinary fields? Investigating researchers in Amsterdam with the revised Publication Pressure Questionnaire, we find that a negative attitude towards the current publication climate is present across academic ranks and disciplinary fields. Postdocs and assistant professors (*M* = 3.42) perceive the greatest publication stress and PhD-students (*M* = 2.44) perceive a significant lack of resources to relieve publication stress. Results indicate the need for a healthier publication climate where the quality and integrity of research is rewarded.

## Introduction

The current state of academia is sometimes referred to as a system affected by hyper-competition [1–3]. This goes hand in hand with strong emphasis on quantitative assessment of scientific output through journal impact factors, citation analyses and the *H*-index [4–6]. The number of publications, citations and grants determine to a large extent the status and recognition of academic researchers [7–10]. Consequently these indicators influence the recruitment, promotion and tenured appointments of researchers [11,12]. This may in turn induce a high level of perceived publication pressure.

In line with Woolf [13], we define perceived publication pressure as the subjective pressure resulting from the feeling that one *has* to publish. In line with work stress literature, strong perceptions of pressure could provoke stress, but need not to when one has many resources available to manage the pressure [14]. Applied to publication pressure: Publication demands and attitude towards the current publication climate determine the perceived pressure, yet pressure can be alleviated by resources like helpful co-authors, involved colleagues or supervisors, and a sense of academic competence [15].

Some degree of publication pressure can be an incentive to produce high quality scientific work [13,16]. Yet, too much publication pressure may have detrimental effects on the scientific enterprise in general and on individual researchers in particular [17]. Excessive publication pressure is associated with poor quality research (and teaching), a decreased willingness to share raw data, less involvement from researchers in public and policy issues, and less academic creativity [16,18–20]. The perceived hypercompetition is thought to lead to less rigorous (“rushing into print”) and less reliable science [1,21,22]. Publication pressure is associated with a greater likelihood to engage in research misbehaviours [23–25]. Lastly, publication pressure is associated with a disproportionate focus on positive and specular findings [21,22,26,27].

Publication pressure may also have detrimental effects on individual researchers. It is linked to a poor research climate and may render academic researchers emotionally exhausted [3,28]. Previous research on publication pressure found junior researchers to experience more publication pressure compared to their senior counterparts [20,23]. Studies investigating publication pressure thus far have mainly included academic researchers from particular disciplines like biomedicine, management and population studies, and included only a subset of academic ranks [16,20,23]. This limits the generalizability of the degree to which researchers perceive publication pressure.

The current study aims to assess whether researchers from all academic ranks (including PhD students) and all disciplinary fields perceive publication pressure. This is important, as differences between academic ranks could signal the need for tailored interventions. Besides, comparing different disciplinary fields may enable us to determine fields that perceive less publication pressure. This may generate new insights in the nature of publication pressure and possible protective factors. Our research question was: What is the level of perceived publication pressure in the four academic institutions in Amsterdam and does the pressure to publish differ between academic ranks and disciplinary fields?

## Materials and methods

### Ethical statement

Our study was ethically reviewed and approved by the Scientific and Ethical Review board of the Faculty of Behavioural and Movement Sciences (Vrije Universiteit Amsterdam).

### Participants

All academic researchers in Amsterdam employed in research for at least one day per week at one of the four academic institutions (Vrije Universiteit Amsterdam, University of Amsterdam and the two Amsterdam University Medical Centers) were eligible to participate. This included PhD students, as in The Netherlands PhD students are employees.

### Procedure

First, we set up a data sharing agreement with all participating institutions to safely obtain the e-mail addresses of their researchers. Second, we sent an information letter inviting all academic researchers in Amsterdam (*n* = 7465) to take part in our study. The information letter contained links to the study protocol (S1 Protocol) and the study’s privacy policy (S1 Appendix). In addition, we included a link to a short non-response questionnaire where we asked researchers to report their academic rank, gender and enquired whether the reason for declining participation resulted from a sense that their data were not protected. For the full non-response questionnaire, see S2 Appendix.

A week later, researchers were invited to complete an online survey. The survey started with an informed consent statement followed by the inclusion check (“Are you currently employed in research for at least one day per week?”) and ended with the demographic items about participants’ academic rank (PhD student, postdoc, assistant professor, associate professor and full professor) and major disciplinary field: biomedicine (consisting of life and medical sciences), natural sciences, social sciences (included both social and behavioural sciences) and humanities (consisting of humanities, language, communication, law and arts). We used Qualtrics (Qualtrics, Provo, UT, USA) to create and distribute the survey, that took approximately 15 minutes to complete. We sent three reminders, each 10 days apart.

### Instruments

We used the revised Publication Pressure Questionnaire (PPQr) to measure publication pressure [15]. The PPQr is a valid and reliable instrument to measure publication pressure and consists of 3 subscales scored on a 5-points Likert scale (‘Totally agree’ = 5, ‘Totally disagree’ = 1). The Publication Stress subscale (6 items—Cronbach’s α = .804) regards the stress a researcher experiences due to the feeling she/he has to publish and includes items such as “I feel forced to spend time on my publications outside office hours”. The Publication Attitude (6 items—Cronbach’s α = .777) subscale reflects researchers’ attitudes towards publication pressure, for example: “Publication pressure harms science”. Finally, the Publication Resources subscale (6 items—Cronbach’s α = .754) consist of factors that can help prevent publication pressure (e.g. feeling of competence, freedom to choose topics of scientific investigation; involved colleagues). A typical item would be: “When working on a publication, I feel supported by my co-authors.”. The full PPQr questionnaire can be found in S3 Appendix.

PPQr subscale scores are computed by taking the average of all items in the subscale. A higher score on all subscales means the researcher perceives publication stress, has a negative attitude towards the publication climate and perceives little publication resources to alleviate publication stress.

The survey contained two other instruments (Survey of Organizational Research Climate [29] and 60 major and minor misbehaviours [30]), but those analyses will be part of another report see [31] and [32]. The interrelations between these concepts will be reported in a separate future paper.

### Statistical analyses

We preregistered our analyses on the Open Science Framework, see osf.io/w4t7u. To summarise: First we calculated overall mean scores for all three subscales and stratified these for academic ranks and disciplinary fields. Second, we assessed whether there were differences between particular academic ranks or disciplinary fields using Bonferroni corrected *F*-tests and Mean Differences (*MD*) with 95% Confidence Intervals (*CI*). Third, we build multivariable regression models to test whether academic rank and disciplinary field were associated with PPQr subscale mean scores. In these regression models, we also looked for evidence of confounding and interaction. Estimates corrected for confounding are provided and instances of interaction were reported. All analyses were conducted using SPSS Statistics.

## Results

### Response rate and inclusion

**Fig 1:**
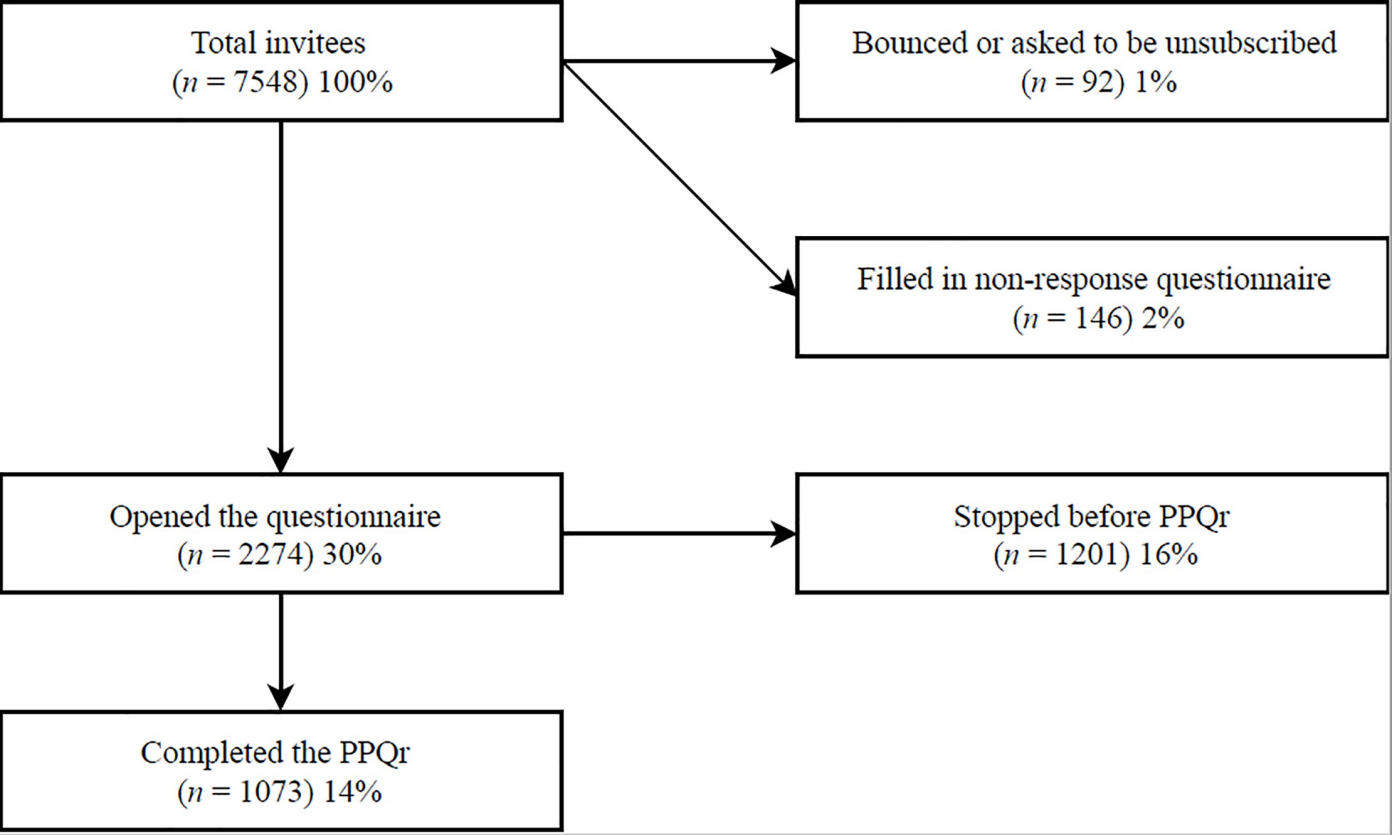
Overview of response rate.

From the 7548 researchers that were invited to participate, 30% (*n* = 2274) followed the link to the online survey. 1073 of invitees filled in the PPQr (response rate = 14%), demographic information is listed in Table 1. About 2% of the invitees filled in the non-response questionnaire. See Fig 1.

**Table 1:** Descriptives of participants, stratified by gender, academic rank and disciplinary field.

		Publication Stress	Publication Attitude	Publication Resources
	*n*	*M* (*SD*)	*M* (*SD*)	*M* (*SD*)
**Males**	441	3.10 (.79)	3.58 (.72)	2.08 (.63)
**Females**	632	3.29 (.79)	3.60 (.65)	2.30 (.61)
**PhD students**	503	3.18 (.80)	3.60 (.67)	2.44 (61)
**Postdocs and assistant professors**	318	3.42 (.74)	3.70 (.63)	2.12 (.55)
Associate and full professors*	216	3.03 (.82)	3.42 (.76)	1.80 (.54)
**Biomedicine**	603	3.16 (.79)	3.60 (.65)	2.24 (.61)
**Natural sciences**	119	3.12 (.80)	3.51 (.77)	2.04 (.68)
**Social sciences**	242	3.32 (.80)	3.60 (.71)	2.24 (.64)
**Humanities**	109	3.42 (.76)	3.58 (.68)	2.16 (.62)
**Total participants**	1073	3.22 (.80)	3.59 (.68)	2.21 (.63)

* 36 participants failed to disclose their academic rank.

Overall, we find academic researchers in our sample to score highest on Attitude (*M* = 3.59). This indicates that the negative attitude towards the publication climate is substantial. There is on average a somewhat lesser degree of Publication Stress (*M* = 3.22) and a relatively small lack of Publication Resources (*M* = 2.21). Stratified and total sample mean scores can be found in Table 1.

### Publication pressure by academic rank

**Fig 2:**
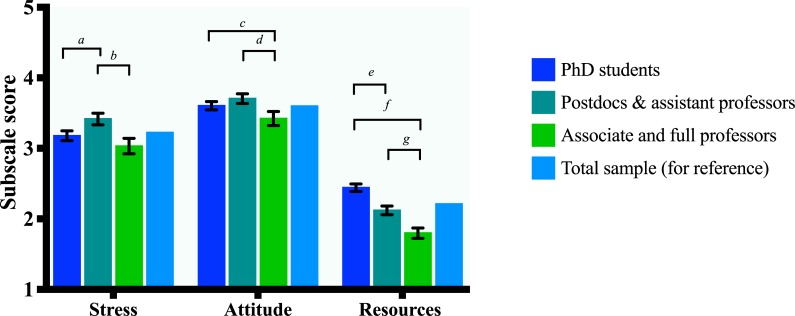
Differences between academic ranks in PPQr subscale scores. Letters denote significant (α *=* .05) *Bonferroni* corrected mean differences (*MD*) between pairs of (denoted by the brackets) academic ranks and error bars express 95% Confidence Intervals (*CI*). *MDs* are corrected for confounders (disciplinary field and gender) if applicable. *N* = 1073. *a*: *MD* = .237, *CI* = (.103, .371) *b*: *MD* = .384, *CI* = (.219, .549) *c*: *MD* = .181, *CI* = (.049, .314) *d*: *MD* = .282, *CI* = (.139, .426) *e*: *MD* = .322, *CI* = (.223, .421) *f*: *MD =* .645, *CI* = (.532, .757) *g*: *MD =* .322 *CI* = (.201, .444).

Pairwise *Bonferroni* and confounding-corrected (disciplinary field and gender) mean differences between academic ranks indicate that postdocs and assistant professors perceive significantly more publication stress than both PhD students and associate and full professors. Besides, both PhD students as well as postdocs and assistant professors have a more negative attitude towards the publication culture compared to full professors. Furthermore, PhD students perceive a significantly greater lack of resources than both postdocs and assistant professors as well as associate and full professors. Finally, postdocs and assistant professors perceive less resources than associate and full professors. See Fig 2. Crude and *Bonferroni* corrected mean differences between pairs of groups can be found in S1 Table. For crude and corrected association models between academic rank and the PPQr subscales, see S2 Table.

### Publication pressure by disciplinary field

**Fig 3:**
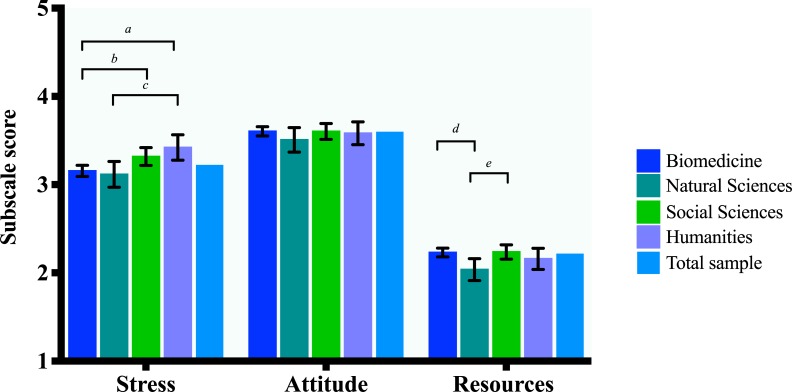
Differences between disciplinary field in PPQr subscale scores. Letters denote significant (α *=* .05) *Bonferroni* corrected mean differences (*MD*) between pairs of (denoted by the brackets) disciplinary fields and error bars express 95% Confidence Intervals (*CI*). *MDs* are corrected for confounders (academic rank and gender) if applicable. *N* = 1073. *a*: *MD* = .297, *CI* = (.080, .515) *b*: *MD* = .202, *CI* = (.042, .363) *c*: *MD* = .318, *CI* = (.040, .596) *d*: *MD* = .204, *CI* = (.048, .359) *e*: *MD* = .210, *CI* = (.036, .384).

Pairwise *Bonferroni* and confounding-corrected (academic rank and gender) mean differences indicate that researchers in the humanities perceive more publication stress than both biomedicine and the natural sciences. Yet the researchers from the social sciences perceive more publication stress than their biomedical colleagues. There were no statistically significant differences between disciplinary fields on attitude scores. Finally, researchers in biomedicine as well as social sciences perceive a significantly greater lack of publication resources than researchers in the natural sciences. See Fig 3. Crude and Bonferroni corrected mean differences between pairs of groups can be found in S1 Table. For crude and corrected disciplinary field association models, see S3 Table.

### Effect modification

We only found effect modification by disciplinary field of the differences between academic ranks’ Publication Resources scores. Differences between PhD students and senior academic researchers in perceived Publication Resourced are greater in natural sciences compared to other disciplinary fields. Stratified results are displayed in Table 2.

**Table 2:** Effect modification from disciplinary field (natural sciences) in the relation between Publication Resources and academic rank^1^.

Resources	*PhD students*	*Postdocs/assistant professors*	*Associate & full professors*
*Natural sciences*	2.36	1.99	1.44
*Biomedical sciences*	2.44	2.16	1.81
*Social sciences*	2.54	2.07	1.84
*Humanities*	2.33	2.25	1.98

^1^ Scores stratified for academic rank and disciplinary field.

### Effect sizes

We found 12 significant differences between pairs of groups and since we performed many statistical tests, it is likely that some of the significant differences are in fact due to chance. To provide the reader with some guidance on which effects are relevant, we calculated effect sizes of each difference. This analysis was not preregistered and thus should be considered exploratory. The effect sizes range from small to very large using Cohen’s effect size criteria [33], see Table 3. To prevent overinterpreting small differences, we will focus further discussion on differences with an effect size of medium or above.

**Table 3:** Significant (*p* < .05) differences with corresponding effect sizes.

Subscale	Group vs. Group	Effect size^1^	Interpretation^2^
**Stress**	PhD students vs. Postdocs < assistant professors	.31	Small
**Stress**	Postdocs & assistant professors < Associate & full professors	.50	Medium
**Attitude**	Associate & full professors < PhD students	.15	Small
**Attitude**	Associate & full professors < Postdocs & assistant professors vs.	.41	Small
**Resources**	Postdocs & assistant professors < PhD students	.55	Medium
**Resources**	Associate & full professors < PhD students	1.09	Very large
**Resources**	Associate & full professors < Postdocs & assistant professors	.59	Medium
**Stress**	Biomedicine < Humanities	.33	Small
**Stress**	Biomedicine < Social sciences	.20	Small
**Stress**	Natural sciences < Humanities	.38	Small
**Resources**	Natural sciences < Biomedicine	.32	Small
**Resources**	Natural sciences < Social sciences	.31	Small

^1^ using Hedges’ *G* computed as: M1−M2SDpooled

^2^ Interpreted based on Cohen [33] where an effect size of .20 is defined as small, .50 is medium, .80 is large and 1.30 is very large.

## Discussion

We assessed the level of perceived publication pressure in the four academic institutions in Amsterdam and whether the pressure to publish differed between academic ranks and disciplinary fields. Overall, there is a negative attitude towards the publication climate. Hence the ‘publish or perish’ mantra from the late 20^th^ century may turn into ‘publish *and* perish’, since even when a researcher publishes reasonably, chances for tenure in academia may still be low [20,34,35]. Below we elaborate on the differences of effect sizes that were medium or above or on those where we found interaction effects [33].

### Academic rank differences

Postdocs and assistant professors perceive most publication stress and have the most negative attitude towards the current publication climate, which is in line with previous studies assessing perceived publication pressure in biomedicine and organisation science [20,23]. This finding seems intuitive as this particular group aims for a (tenured) position and promotion criteria are to a large extent based on quantitative publication indicators. Associate and full professors have already an established position, and consequently may perceive less publication pressure. PhD candidates’ likelihood of successfully defending their thesis is usually not dependent on the number of publications. This may explain why their publication pressure level is somewhat lower. Besides, some PhD students may not aspire an academic career and will therefore presumably perceive less publication pressure then their colleagues who wish to pursue an academic career.

However, PhD candidates perceive the greatest lack of resources. This is both alarming and understandable. Arguably, PhD students are inexperienced in handling difficulties that may arise when working on a publication. The same holds for starting postdocs. Consequently, junior researchers could benefit most from supportive colleagues and supervisors. Unfortunately mentoring may be suboptimal [30,36,37].

### Disciplinary field differences

Differences between disciplinary fields were significant but small. Hence, we focus here on the interaction between disciplinary field and academic rank in perceived Resources. Researchers from the natural sciences perceived most publication resources, which may be due to their typical organisation (large) research teams where collaboration is vital for discovery. However, PhD students in the natural sciences perceive a lack of resources that is similar to PhD students from the other disciplinary fields. It may be that insufficient mentoring in the publication process makes them feel incompetent and insecure.

### Strengths

This is the first study that comprehensively measured publication pressure with a validated measurement instrument. The three dimensions, stress, attitude and resources, respectively, are meaningful components when conceptualising publication pressure. Also, these three dimensions are sufficiently distinctive in the data reported here [15].

Second, this is the first study to investigate publication pressure across academic ranks and disciplinary fields. It can serve as a benchmark for future studies. We managed to include a substantial number of participants in our study that increases the reliability of the differences found.

### Limitations

Our study also has some limitations we would like to address. First, we have a relatively low completion rate (14%) which may be an indication of response bias, although our completion rate is similar to other web-based surveys [38]. Only 2% of our invitees filled in the non-response questionnaire, which we consider to be too little to assess whether non-responders differed from responders. Perhaps invitees chose not to respond because they were to focused on their publications, leading to an underestimation. Related, simply mentioning that our study investigated the publication culture could have prompted negative connotations with the publication culture, as it has not gone unnoticed in the public debate in The Netherlands.

To assess the representativeness of our sample, we first looked into the population characteristics. In our sample, 56% of completers indicated working in the biomedical field, whereas 53% of our invitees was employed at one of the Amsterdam University Medical Centers, indicating a small overrepresentation from biomedicine.

Statistics on PhD students employed at both universities in Amsterdam indicated that PhD students make up 30% of the academic workforce, whereas PhD students formed 41% of our sample. Likewise, 44% of academic researchers in Amsterdam is female, yet women made up 57% of our sample, indicating overrepresentation of both PhD students and women.

However, we corrected for the potential gender bias by adjusting our estimates for confounding variables. Besides, we found no effect modification from gender. To conclude, it is unlikely that the selectivity of our sample biased our results.

To assess possible response bias, we conducted a wave analysis. We used late responders–those who responded after the last reminder–as a proxy for nonresponders and compared these to early responders–those who responded after the initial invitation–as described by Phillips’ and colleagues [39]. Differences were .13, .07 and .02 for Stress, Attitude and Resources, respectively. These differences were then multiplied by the proportion of non-responders, in our case 86%. Consequently, the non-response bias was .11, .07 and .02 for Stress, Attitude and Resources, respectively. This was found to be small compared to the difference that we observed between the subgroups which ranged from .18 to .65. It is therefore unlikely that non-response affected our conclusions.

Besides, the PPQr focuses exclusively on publication pressure. However, research is not conducted in a vacuum and if teaching or other professional duties put excessive demands on a researcher, then naturally there is less time left for publishing, which could lead to elevated levels of publication pressure. This can be labelled as role-conflict: you are expected to meet different obligations, i.e. teaching, research, and professional duties, in a naturally limited amount of time [40]. How much stress is due to *just* publication pressure is unclear (see also [15]).

Relatedly, universities have been subject to neoliberal and Taylorist reforms that were—in a nutshell—intended to make universities more competitive and were accompanied with an excessive focus on researchers’ performance management, perhaps at the expense of traditional hallmarks of the academia such as teaching and collegiality [41,42]. A full review of Neoliberal and Taylorist reforms in academia is beyond the scope of this paper (the reader is referred to Lorenz’ excellent paper [43] that includes specific examples of reforms in Dutch academia) but it seems feasible to reason that publication pressure is one of its consequences, although the exact relation has, to our knowledge, not been studied systematically.

Finally, since this is the first study conducted with the PPQr, it’s rather difficult to interpret the absolute levels and differences in publication pressure we found.

### Future research

Future work should aim to explore if the differences we found generalise internationally. Publication climates in the USA and Asian countries may be different as their funding systems greatly differ [2,44,45]. However, the same could apply to closer examples such as Germany and Belgium, as those funding systems are also somewhat different from those in the Netherlands. Interestingly, a study with an previous version of the PPQ found Flemish biomedical researchers to experience more publication pressure than their Dutch colleagues [23]. Besides, it will be informative to study publication pressure longitudinally to see if it is associated with burn-out and research misbehaviour. Finally, it would be intriguing to investigate qualitatively what it means for researchers to experience high publication pressure and how it impacts their academic work.

### Conclusions

Taken together, publication pressure concerns researchers from all disciplinary fields and seems to be a particularly detrimental stressor for postdocs and assistant professors. In addition, PhD students perceive a significant lack of resources that may hamper their development into responsible researchers. The amount of resources is perceived to be better among researchers from the natural sciences, but PhD students in this disciplinary field nevertheless would also benefit from more support from their senior colleagues. Our findings emphasize the need to move the debate forward towards a healthy publication climate, where researchers are incentivised to focus on the quality and the integrity of their publications and feel supported to conduct responsible research.

## Supporting information

S1 Protocol Academic Research Climate Amsterdam study protocol. (DOCX)

S1 Appendix Privacy policy. (DOCX)

S2 Appendix Non-response questionnaire. (DOCX)

S3 Appendix Full PPQr questionnaire with corresponding Cronbach’s alphas. Items are scored on a 5-points Likert scale where 1 = “Totally disagree” and 5 = “Totally agree”.
(DOCX)

S1 Table Crude and Bonferroni corrected mean differences (MD) with 95% Confidence Intervals (CI) between academic ranks and disciplinary fields. (DOCX)

S2 Table Crude (in italics) and corrected academic rank association models. Interpretation: For gender, male is the reference category. For the disciplinary field dummies, humanities is coded as the reference category. For academic rank dummies, this is associate and full professors. PhDs = PhD students, assis prof = assistant professors, asso prof = associate professors, full prof = full professors.
(DOCX)

S3 Table Crude (in italics) and corrected disciplinary field association models. Interpretation: For gender, male is the reference category. For the disciplinary field dummies, humanities is coded as the reference category. For academic rank dummies, this is associate and full professors. PhDs = PhD students, assis prof = assistant professors, asso prof = associate professors, full prof = full professors.
(DOCX)
