# Supplementary material for: Perceived publication pressure in Amsterdam: Survey of all disciplinary fields and academic ranks
Source: PLoS One. 2019 Jun 19;14(6):e0217931. doi: 10.1371/journal.pone.0217931 (PMC6583945; doi:10.1371/journal.pone.0217931)
Supplement: S2 Appendix — (DOCX) [file pone.0217931.s003.docx]

**Non-response survey**

NR1: We realize that you prefer not to participate in this survey.

Surveys may suffer from non-response that is non-random. To help us get a clearer picture of which type of persons prefer not to participate, we should like to learn only your rank, sex and perception of this survey as 100% safe. Would you be willing to share only that information? (Yes/no)
NR2: What is your gender? (Male/Female)

NR3: What is your academic rank? (PhD student/ Postdoc / Assistant professor / Associate professor/ Full professor / Other)

NR4: To what extent does the following statement apply to you?
I chose not to participate because the I did not feel like my data were protected (Totally disagree/Disagree/Agree nor disagree/Agree/Totally agree)
