# Supplementary material for: Perceived publication pressure in Amsterdam: Survey of all disciplinary fields and academic ranks
Source: PLoS One. 2019 Jun 19;14(6):e0217931. doi: 10.1371/journal.pone.0217931 (PMC6583945; doi:10.1371/journal.pone.0217931)
Supplement: S3 Appendix — Items are scored on a 5-points Likert scale where 1 = “Totally disagree” and 5 = “Totally agree”. (DOCX) [file pone.0217931.s004.docx]

**S3 Appendix. PPQr subscales with corresponding Cronbach’s alphas and items.**

| **Subscales (Cronbach’s *α*)** | **Items** |
| --- | --- |
| **Stress (.83)** | *N* = 6 |
|  | I experience stress at the thought of my colleagues' assessment of my publications output. |
|  | I feel forced to spend time on my publications outside office hours. |
|  | I cannot find sufficient time to work on my publications. |
|  | I have no peace of mind when working on my publications. |
|  | I can combine working on my publications with my other tasks. |
|  | At home, I do not feel stressed about my publications. |
| **Attitude (.80)** | *N* = 6 |
|  | The current publication climate puts pressure on relationships with fellow-researchers. |
|  | I suspect that publication pressure leads some colleagues (whether intentionally or not) to cut corners. |
|  | In my opinion the pressure to publish scientific articles has become too high. |
|  | My colleagues judge me mainly on the basis of my publications. |
|  | Colleagues maintain their administrative and teaching skills well, despite publication pressure. |
|  | Publication pressure harms science. |
| **Resources (.76)** | *N* = 6 |
|  | When working on a publication, I feel supported by my co-authors. |
|  | When I encounter difficulties when working on a publication, I can discuss these with my colleagues. |
|  | I have freedom to decide about the topics of my publications. |
|  | When working on a publication, many decisions about the content of the paper are outside my control. |
|  | I cannot cope with all aspects of publishing my papers. |
|  | I feel confident in the interaction with co-authors, reviewers and editors. |
