# Supplementary material for: Perceived publication pressure in Amsterdam: Survey of all disciplinary fields and academic ranks
Source: PLoS One. 2019 Jun 19;14(6):e0217931. doi: 10.1371/journal.pone.0217931 (PMC6583945; doi:10.1371/journal.pone.0217931)
Supplement: S1 Table — (DOCX) [file pone.0217931.s005.docx]

**S1 Table. Crude and *Bonferroni* corrected mean differences (*MD*) with 95% Confidence Intervals (CI) between academic ranks and disciplinary fields.**

| **PPQr Subscale** | **Pairs compared** | ***MD* Crude** | **CI Crude** | ***MD Bonferroni* corrected** | **CI *Bonferroni* corrected** |
| --- | --- | --- | --- | --- | --- |
|  | *Academic rank* |  |  |  |  |
| Stress | PhD students vs. postdocs/assis. prof | .237 | (.128, .347) | .237 | (.102, .373) |
|  | Postdocs/assis. prof vs. asso./full professors | .384 | (.250, .518) | .384 | (.217, .551) |
|  | PhD students vs. asso./full professors | .147 | (.018, .256) | *NS* | *NS* |
| Attitude | PhD students vs. postdocs/assis. prof | .101 | (.009, .193) | *NS* | *NS* |
|  | PhD students vs. asso./full professors | .181 | (.070, .293) | .181 | (.049, .314) |
|  | Postdocs/assis. prof vs. asso./full professors | .282 | (.164, .401) | .282 | (.139, .426) |
| Resources | PhD students vs. postdocs/assis. prof | .322 | (.239, .405) | .322 | (.223, .422) |
|  | PhD students vs. asso./full professors | .645 | (.550, .739) | .645 | (.532, .758) |
|  | Postdocs/assis. prof vs. asso./full professors | .322 | (.227, .418) | .322 | (.200, .445) |
|  | *Disciplinary field* |  |  |  |  |
| Resources | Biomedicine *vs.* natural sciences | .204 | (.081, .326) | .204 | (.038, .370) |
|  | Social sciences *vs.* natural sciences | .200 | (.056, .343) | .200 | (.014, .385) |
| Stress | Social sciences *vs.* biomedicine | .163 | (.044, .282) | .163 | (.003, .322) |
|  | Humanities *vs.* biomedicine | .265 | (.104, .426) | .265 | (.047, .483) |
|  | Humanities *vs.* natural sciences | .304 | (.100, .508) | .304 | (.026, .582) |
|  | Social sciences *vs.* natural sciences | .202 | (.026, .378) | *NS* | *NS* |
