# Supplementary material for: Perceived publication pressure in Amsterdam: Survey of all disciplinary fields and academic ranks
Source: PLoS One. 2019 Jun 19;14(6):e0217931. doi: 10.1371/journal.pone.0217931 (PMC6583945; doi:10.1371/journal.pone.0217931)
Supplement: S2 Table — Interpretation: For gender, male is the reference category. For the disciplinary field dummies, humanities is coded as the reference category. For academic rank dummies, this is associate and full professors. PhDs = PhD students, assis prof = assistant professors, asso prof = associate professors, full prof = full professors. (DOCX) [file pone.0217931.s006.docx]

**S2 Table. Crude (in *italics)* and corrected academic rank association models.**

|  | ***β*** | ***SE*** | ***CI*** |
| --- | --- | --- | --- |
| **Dependent variable: Publication Stress** |  |  |  |
| *Intercept* | 3.032 | .054 | (2.926, 3.137) |
| *Rank dummy 1 (PhDs vs. asso & full prof)* | .147 | .064 | (.021, .273) |
| *Rank dummy 2 (Postdocs & assis prof. vs. asso & full prof)* | .384 | .070 | (.248, 520) |
|  |  |  |  |
| Intercept | 3.357 | .091 | (3.179, 3.535) |
| Rank dummy 1 (PhDs vs. asso & full prof) | .120 | .066 | (-.010, .250) |
| Rank dummy 2 (Postdocs & assis prof. vs. asso & full prof) | .344 | .071 | (.205, .483) |
| Discipline dummy 1 (biomedicine vs. humanities) | -.301 | .083 | (-.464, -.138) |
| Discipline dummy 2 (natural sciences vs. humanities) | -.292 | .107 | (-.502, -.082) |
| Discipline dummy 3 (social sciences vs. humanities) | -.165 | .093 | (-.347, .017) |
| Gender (male vs. female) | -.146 | .052 | (-.249, -.044) |
| **Dependent variable: Publication Attitude** |  |  |  |
| Intercept | 3.422 | .046 | (3.332, 3.513) |
| Rank dummy 1 (PhDs vs. asso & full prof) | .181 | .055 | (.073, .289) |
| Rank dummy 2 (Postdocs & assis prof. vs. asso & full prof) | .282 | .060 | (.165, .400) |
| **Dependent variable: Publication Resources** |  |  |  |
| *Intercept* | 1.798 | .039 | (1.720, 1.875) |
| *Rank dummy 1 (PhDs vs. asso & full prof)* | .645 | .047 | (.552, .737) |
| *Rank dummy 2 (Postdocs & assis prof. vs. asso & full prof)* | .322 | .051 | (.222, .423) |
|  |  |  |  |
| Intercept | 1.977 | .095 | (1.790, 2.164) |
| Rank dummy 1 (PhDs vs. asso & full prof) | .351 | .138 | (.081, .622) |
| Rank dummy 2 (Postdocs & assis prof. vs. asso & full prof) | .268 | .139 | (-.005, .541) |
| Discipline dummy 1 (biomedicine vs. humanities) | -.165 | .108 | (-.378, .048) |
| Discipline dummy 2 (natural sciences vs. humanities) | -.541 | .147 | (-.830, -.252) |
| Discipline dummy 3 (social sciences vs. humanities) | -.138 | .144 | (-.421, .146) |
| Rank dummy 1 * Discipline dummy 1 | .274 | .151 | (-.022, .570) |
| Rank dummy 1 * Discipline dummy 2 | .575 | .195 | (.192, .958) |
| Rank dummy 1 * Discipline dummy 3 | .346 | .185 | (-.016, .709) |
| Rank dummy 2 * Discipline dummy 1 | .081 | .156 | (-.225, .387) |
| Rank dummy 2 * Discipline dummy 2 | .292 | .203 | (-.106, .689) |
| Rank dummy 3 * Discipline dummy 2 | -.040 | .185 | (-.404, .323) |
