# Supplementary material for: Perceived publication pressure in Amsterdam: Survey of all disciplinary fields and academic ranks
Source: PLoS One. 2019 Jun 19;14(6):e0217931. doi: 10.1371/journal.pone.0217931 (PMC6583945; doi:10.1371/journal.pone.0217931)
Supplement: S3 Table — Interpretation: For gender, male is the reference category. For the disciplinary field dummies, humanities is coded as the reference category. For academic rank dummies, this is associate and full professors. PhDs = PhD students, assis prof = assistant professors, asso prof = associate professors, full prof = full professors. (DOCX) [file pone.0217931.s007.docx]

**S3 Table. Crude (in italics) and corrected disciplinary field association models.**

|  | ***β*** | ***SE*** | ***CI*** |
| --- | --- | --- | --- |
| **Dependent variable: Publication Stress** |  |  |  |
| *Intercept* | 3.420 | .076 | (.3271, 3.570) |
| *Discipline dummy 1 (biomedicine vs. humanities)* | -.265 | .083 | (-.427, -.103) |
| *Discipline dummy 2 (natural sciences vs. humanities)* | -.304 | .105 | (-.511, -.098) |
| *Discipline dummy 3 (social sciences vs. humanities)* | -.102 | .091 | (-.282, .077) |
|  |  |  |  |
| Intercept | 3.357 | .091 | (3.179, 3.535) |
| Discipline dummy 1 (biomedicine vs. humanities) | -.301 | .083 | (-.464, -.138) |
| Discipline dummy 2 (natural sciences vs. humanities) | -.292 | .107 | (-.502, -.082) |
| Discipline dummy 3 (social sciences vs. humanities) | -.165 | .093 | (-.347, .017) |
| Rank dummy 1 (PhDs vs. asso & full prof) | .120 | .066 | (-.010, .250) |
| Rank dummy 2 (Postdocs & assis prof. vs. asso & full prof) | .344 | .071 | (.205, .483) |
| Gender (male vs. female) | -.146 | .052 | (-.249, -.044) |
| **Dependent variable: Publication Resources** |  |  |  |
| *Intercept* | 2.159 | .061 | (2.039, 2.279) |
| *Discipline dummy 1 (biomedicine vs. humanities)* | .082 | .066 | (-.048, .212) |
| *Discipline dummy 2 (natural sciences vs. humanities)* | -.122 | .084 | (-.287, .043) |
| *Discipline dummy 3 (social sciences vs. humanities)* | .078 | .073 | (-.066, .221) |
|  |  |  |  |
| Intercept | 1.977 | .095 | (1.790, 2.164) |
| Discipline dummy 1 (biomedicine vs. humanities) | -.165 | .108 | (-.378, .048) |
| Discipline dummy 2 (natural sciences vs. humanities) | -.541 | .147 | (-.830, -.252) |
| Discipline dummy 3 (social sciences vs. humanities) | -.138 | .144 | (-.421, .146) |
| Rank dummy 1 (PhDs vs. asso & full prof) | .351 | .138 | (.081, .622) |
| Rank dummy 2 (Postdocs & assis prof. vs. asso & full prof) | .268 | .139 | (-.005, .541) |
| Discipline dummy 1 * Rank dummy 1 | .274 | .151 | (-.022, .570) |
| Discipline dummy 1 * Rank dummy 2 | .081 | .156 | (-.225, .387) |
| Discipline dummy 2 * Rank dummy 1 | .575 | .195 | (.192, .958) |
| Discipline dummy 2 * Rank dummy 2 | .292 | .203 | (-.106, .689) |
| Discipline dummy 3 * Rank dummy 1 | .346 | .185 | (-.016, .709) |
| Discipline dummy 3 * Rank dummy 2 | -.040 | .185 | (-.404, .323) |
